# Endoplasmic Reticulum Stress Sensor IRE1α Enhances IL-23 Expression by Human Dendritic Cells

**DOI:** 10.3389/fimmu.2017.00639

**Published:** 2017-06-19

**Authors:** Saioa Márquez, José Javier Fernández, Eli Terán-Cabanillas, Carmen Herrero, Sara Alonso, Alicia Azogil, Olimpio Montero, Takao Iwawaki, Juan R. Cubillos-Ruiz, Nieves Fernández, Mariano Sánchez Crespo

**Affiliations:** ^1^Departamento de Bioquímica y Biología Molecular, Facultad de Medicina, Universidad de Valladolid, Valladolid, Spain; ^2^Department of Obstetrics and Gynecology, Weill Cornell Medical College, New York, NY, United States; ^3^Sandra and Edward Meyer Cancer Center, Weill Cornell Medical College, New York, NY, United States; ^4^Unidad Académica de Ciencias de la Nutrición y Gastronomía, Universidad Autónoma de Sinaloa, Culiacán, México; ^5^Instituto de Biología y Genética Molecular, CSIC-Universidad de Valladolid, Valladolid, Spain; ^6^Centro para el Desarrollo de la Biotecnología, CSIC, Parque Tecnológico de Boecillo, Valladolid, Spain; ^7^Division of Cell Medicine, Medical Research Institute, Kazanawa Medical University, Ishikawa, Japan

**Keywords:** glycolysis, C/EBPβ, 2-deoxy-d-glucose, fungal patterns, IL-23, lactate, unfolded protein response, XBP1

## Abstract

Human monocyte-derived dendritic cells (DCs) exposed to pathogen-associated molecular patterns (PAMPs) undergo bioenergetic changes that influence the immune response. We found that stimulation with PAMPs enhanced glycolysis in DCs, whereas oxidative phosphorylation remained unaltered. Glucose starvation and the hexokinase inhibitor 2-deoxy-d-glucose (2-DG) modulated cytokine expression in stimulated DCs. Strikingly, *IL23A* was markedly induced upon 2-DG treatment, but not during glucose deprivation. Since 2-DG can also rapidly inhibit protein N-glycosylation, we postulated that this compound could induce IL-23 in DCs via activation of the endoplasmic reticulum (ER) stress response. Indeed, stimulation of DCs with PAMPs in the presence of 2-DG robustly activated inositol-requiring protein 1α (IRE1α) signaling and to a lesser extent the PERK arm of the unfolded protein response. Additional ER stressors such as tunicamycin and thapsigargin also promoted IL-23 expression by PAMP-stimulated DCs. Pharmacological, biochemical, and genetic analyses using conditional knockout mice revealed that IL-23 induction in ER stressed DCs stimulated with PAMPs was IRE1α/X-box binding protein 1-dependent upon zymosan stimulation. Interestingly, we further evidenced PERK-mediated and CAAT/enhancer-binding protein β-dependent *trans*-activation of *IL23A* upon lipopolysaccharide treatment. Our findings uncover that the ER stress response can potently modulate cytokine expression in PAMP-stimulated human DCs.

## Introduction

Activation of immune cells induces metabolic changes to control microbial invasion. Glycolysis is needed for many functional tasks in activated macrophages, dendritic cells (DCs), and Th1 and Th17 lymphocytes, e.g., cytokine production and the development of innate immune memory. Consequently, accumulation of lactate and extracellular acidification are prevalent in immune cells stimulated with pathogen-associated molecular patterns (PAMPs). In addition to the production by immune cells, lactate generated by neighbor client cells is a signal of tissue damage that impacts the macrophage response (1). Intracellular lactate stabilizes HIF1α protein (2, 3). An effect shared by other soluble factors found in tumoral and inflammatory milieus. For instance, succinate (4), pyruvate (2), adenosine (5), and hydrogen ions (6, 7). Extracellular lactate can activate the membrane receptor GPR81, which transduces extracellular signals through heterotrimeric G proteins in some cell types (8). A property shared by succinate, which enhances the function of some elements of the TLR family by engaging the G protein-coupled receptor GPR91 (9).

This plethora of metabolic changes entails the necessity of control mechanisms to avoid endoplasmic reticulum (ER) stress and cell death. Given that the unfolded protein response (UPR) is the main mechanism evolved to face this risk, it seems likely that elements of the UPR could support the metabolic and transcriptional rewiring elicited by the interaction of phagocytes with PAMPs. Among the components of the UPR, inositol-requiring protein 1α (IRE1α) is critical to DCs activity since its endoribonuclease domain enables the activation of the X-box binding protein 1 (XBP1) transcription factor (10), which has been demonstrated to control DCs function under diverse physiological and pathological conditions (11). XBP1 also sustains the production of TNFα and IL-6 in macrophages upon TLR-driven activation (12). The clinical relevance of these findings is highlighted by the recent description of the role of XBP1 in the control of antitumor immunity and in the disruption of DCs homeostasis (13), as well as by sound hypotheses associating ER stress caused by HLA-B27 misfolding with IL-23 production in a rat model of spondyloarthritis (14) and the involvement of the IL-23/IL-17 axis in triggering tumor-elicited inflammation and tumor growth (15). These findings have encouraged the search of mechanisms associating the UPR with the transcriptional regulation of *IL23A*, the gene encoding the p19 chain of IL-23. One report described the involvement of C/EBP homologous protein (CHOP), a transcription factor activated in the UPR, when ER stress occurred in the presence of TLR agonists (16), and another report showed an increase of *IL23A* transcription upon inhibition of ataxia telangiectasia mutated kinase that correlated with an increase of the mRNA of spliced *XBP1* (17). However, other studies have shown inhibition of the activating transcription factor 4 (ATF4)/CHOP branch of the UPR by TLR signaling (18, 19) and the actual involvement of ATF2 and NF-κB in the *trans*-activation of *IL23A* in the absence of a definite UPR activation (20, 21). Given the role of glycolysis during immune cell activation, 2-deoxy-d-glucose (2-DG) has been used as a surrogate of glucose deprivation in experimental studies. 2-DG is phosphorylated by hexokinase and this leads to the competitive inhibition of the enzyme (22). The use of 2-DG has disclosed the central function of glucose metabolism in bacterial and viral infection by showing in the latter model the lethality associated with 2-DG treatment by a mechanism involving type I IFN signaling and *Ddit3*/CHOP (23). However, 2-DG is not only a glucose mimetic, it is also the product resulting from the loss of the hydroxyl group attached at carbon-2 of mannose. Given that mannose is present in bodily fluids at much lower concentrations than glucose, 2-DG exerts a stronger competition for mannose metabolism than that exerted on glucose (24). This impinges on reactions using mannose as a substrate, including the formation of the dolichol-linked oligosaccharide Glc3-Man9-GlcNAc2 involved in the initial steps of N-linked glycosylation. The functional relevance of this mechanism has been underscored in several models. For instance, 2-DG inhibits cell surface expression of NKG2D ligands (25), and reduces N-glycosylation and activity of COX-2 (26), a key enzyme for prostaglandin metabolism that contains four glycosylated asparagines, three of which are located in the active center and required for proper protein folding and catalysis (27). In this study, we analyzed the bioenergetic rewiring of human DCs stimulated with the fungal glucan zymosan and bacterial lipopolysaccharide (LPS) and investigated how the UPR modulates cytokine expression. Here, we show (i) a rapid enhancement of glycolysis and the maintenance of oxidative phosphorylation in human DCs; (ii) robust activation of the IRE1α/XBP1 arm of the UPR by 2-DG in the presence of PAMPs, whereas the sole addition of 2-DG induced *DDIT3*/CHOP expression; and (iii) a robust enhancement of *IL23A trans*-activation by 2-DG associated with the induction of the UPR that shows some differences as to the arms triggered by the different PAMPs. An IRE1α/XBP1-dependent mechanism is involved in response to zymosan, whereas the response to LPS depends on IRE1α and PERK, as well as on the recruitment of CAAT/enhancer-binding protein β (C/EBPβ). Taken together, these findings demonstrate a strong influence of the UPR on the cytokine-signature elicited by stimulation of DCs.

## Materials and Methods

### Cells and Reagents

Dendritic cells were obtained from human mononuclear cells collected from pooled buffy coats of healthy donors provided by Centro de Hemoterapia de Castilla y León. The differentiation of monocytes was carried out in the presence of GM-CSF and IL-4 for 5 days. Culture was carried out in RPMI 1640 medium containing 11.1 mM d-glucose, 4 mM L-glutamine, and 1 mM pyruvate. 10% FBS was maintained during the differentiation process and reduced to 2% prior to the initiation of the experiments. Glucose starving experiments were carried out in BioWhittaker^®^ RPMI 1640 medium, containing l-glutamine, without glucose. These conditions were used to replicate the experimental setting used in the Seahorse assays. For the characterization of the cell population as DCs, the expression of CD11c, CD40, and CD86 was assayed. Briefly, cells were centrifuged for 5 min at 350*g* and resuspended in PBS. Ab was added at the concentration of 0.5 µg for 5 × 10^5^ cells and incubated for 45 min at 4°C. When the Ab was labeled with fluorochrome, cells were washed and fixed in 1% formaldehyde. In the case of non-labeled Ab, indirect immunofluorescence was carried out using a labeled secondary Ab before washing and formaldehyde fixation steps. Isotype-matched irrelevant Ab was used as control. The analysis was performed in a Gallios Flow Cytometer. At least 10,000 cells were analyzed per sample. Kaluza software version 1.1 (Beckman Coulter) was used for quantitative data analysis. *Ern1*^f/f^Vav1-Cre mice were obtained by Dr. Cubillos-Ruiz through a collaboration with Prof. Takao Iwawaki. Bone marrow from the femora and tibiae was used to obtain bone marrow-derived dendritic cells (BMDCs) from C57BL/6 [wild type (WT)], *Ern1*^f/f^, and *Ern1*^f/f^Vav1-Cre mouse by incubation in media supplemented with 20 ng/mL recombinant murine GM-CSF. Cells were harvested at day 7 of differentiation and used directly for subsequent studies. The inhibitors of the ribonuclease activity of IRE1α, MK8866, and 4μ8C were a gift from Dr. John Patterson from MannKind Corporation, Valencia, CA, USA (28) and purchased to Tocris, respectively. The PERK inhibitors GSK2606414 and AMG PERK 44 (29, 30) were from Sellenckchem and Tocris, respectively. Tunicamycin was from Calbiochem. 2-DG, LPS, thapsigargin, and zymosan were from Sigma.

### Ethics Statement

The study was approved by the Bioethical Committee of the Spanish Council of Research (CSIC) and the written informed consent of all healthy donors was obtained at Centro de Hemoterapia y Hemodonación de Castilla y León Biobank. The participants received written consent according to the regulations of the Biobank and the researchers received the samples in an anonymous way. The process is documented by the Biobank authority according to the specific Spanish regulations. The animal experiments were carried out with permission of the local authority and conform to institutional standards. The ethics committee approved this procedure before starting the study.

### Bioenergetic Studies

Bioenergetic analyses of human DCs were carried out using the Seahorse Bioscience XF24 Extracellular Flux analyzer (Seahorse Bioscience). DCs were stimulated and either adhered immediately to polyornithine-coated Seahorse plates or after 4 h of incubation. To normalize the recording of data, results are referred to μg of protein. Extracellular acidification rate (ECAR) and oxygen consumption rate (OCR) were analyzed according to the XF Cell Mito Stress Test kit protocol in XF media (non-buffered RPMI medium 1640 containing 11.1 mM glucose, 4 mM l-glutamine, and 1 mM sodium pyruvate), under basal conditions, and in response to 1 µM oligomycin, 1.5 µM FCCP, and 100 nM rotenone plus 1 µM antimycin A.

### Lactate Assay

Lactate was assayed using a colorimetric test in cell supernatants centrifuged at 13,000*g* for 10 min to remove insoluble material and deproteinized by filtration with 3 kDa MWCO spin filters to remove lactate dehydrogenase. The soluble fraction was directly assayed using a Lactate Assay Kit II from Sigma.

### *XBP1* Splicing Assay

This was carried out by RT-PCRs with primers spanning the unspliced regions (Table 1). The PCR conditions were 5 min at 95°C (hot start), 45 cycles of denaturation at 95°C for 15 s, annealing at 60°C for 20 s and elongation at 72°C for 1 min. Final extension was carried out at 72°C for 5 min. Gel electrophoresis was carried out in 3% agarose and spliced *XBP1* and unspliced *XBP1* bands visualized by GelRed™ staining.

**Table 1:** Primers for Q-PCR used in (A) human samples and (B) murine samples.

A. Primers for Q-PCR used in human samples	
*IL1B* FWD	5′-ATGATGGCTTATTACAGTGGCAA-3′
*IL1B* REV	5′-GTCGGAGATTCGTAGCTGGA-3′
*IL10* FWD	5′-GAGAACAGCTGCACC CAC TT-3′
*IL10* REV	5′-GGCCTTGCTCTTGTT TTCAC-3′
*IL12A* FWD	5′-GAGGCCTGTTTACCATTGGA-3′
*IL12A* REV	5′-TCAAGGGAGGATTTTTGTGG-3′
*IL12B* FWD	5′-CATGGGCCTTCATGCTATTT-3′
*IL12B* REV	5′-TTT GCATTG TCAGGTTTCCA-3′
*IL23A* FWD	5′-CATGGGCCTTCATGCTATTT-3′
*IL23A* REV	5′-TTT GCATTG TCAGGTTTCCA-3′
*TNFA* FWD	5′-GTTGTAGCAAACCCTCAAGC-3′
*TNFA* REV	5′-TTGAAGAGGACCTGGGAGTA-3′
*DDIT3/*CHOP FWD	5′-GCAGAGATGGCAGCTGAGTC-3′
*DDIT3/*CHOP AS	5′-AGCCAAGCCAGAGAAGCAGGGT-3′
*PKM1* FWD	5′-GCATCATGCTGTCTGGAGAA-3′
*PKM1* REV	5′-AACTATCAAAGCTGCTGCTA-3′
*PKM2* FWD	5′-CTATCCTCTGGAGGCTGTGC-3′
*PKM2* REV	5′-ACGATTATGGCCCCACTGCA-3′
*XBP1* FWD	5′-TAAGACAGCGCTTGGGGATGGA-3′
*XBP1* REV	5′-ATACCGCCAGAATCCATGGGGA-3′
*GFPT1* FWD	5′-AATGCTGGTCCTGAGATTGG-3′
*GFPT1* REV	5′-TTGATTTTCAGTGCCCCTTC-3′
*GAPDH* FWD	5′-GTCAGTGGTGGACCTGACCT-3′
*GAPDH* REV	5′-AGGGGAGATTCAGTGTGGTG-3′

**B. Primers for Q-PCR used in murine samples**	

*sXbp*1 FWD	5′-AAGAACACGCTTGGGAATGG-3′
*sXbp*1 REV	5′-CTGCACCTGCTGCGGAC-3′
*Ddit3* FWD	5′-GTCCCTAGCTTGGCTGACAGA-3′
*Ddit3* REV	5′-TGGAGAGCGAGGGCTTTG-3′
*Il23a FWD*	5′-AGGGAACAAGATGCTGGATT-3′
*IL23aREV*	5′-AGTAGATTCATATGTCCCGCT-3′

### Pyruvate Kinase M (PKM) mRNA Expression

Pyruvate kinase M isoforms are generated by alternative splicing of mutually exclusive exons and differ in that *PKM1* mRNA contains exon 9 and lacks exon 10, whereas *PKM2* mRNA includes exon 10 and lacks exon 9. RT-PCRs were carried out with primers designed in exon 8 and 11, to yield a 218 bp *PKM1* mRNA and a 183 bp *PKM2* mRNA. This allows the quantitative assay of each isoform and the identification of the PCR amplicon by digestion with *Stu*I, which recognizes an AGGCT sequence in exon 10 and yields a 149 and a 34 bp product in *PKM2*.

### Western Blotting

Proteins were separated by electrophoresis in SDS/PAGE and transferred to nitrocellulose membranes. The membranes were used for the immunodetection of COX-2 (Santa Cruz sc-1745), CHOP (Santa Cruz sc-793), elongation initiation factor 2α (eIF2α, Cell Signaling #5324), P-Y705-STAT3 (Cell Signaling #9131), STAT3 (Cell Signaling #9132), PKM2 (Cell Signaling #3198S), IL-1β (Cell Signaling #12242), histone H3 (abcam ab1791), acetyl-K9-histone H3 (Millipore #4-1003;), P-S10-histone H3 (Millipore #04-817), spliced XBP1 (sXBP1; BioLegend #619501), ATF6 (abcam ab11909), HIF1α (Novus Biologicals NBP1-19779), and P-S52-eIF2α (Invitrogen #44-728G). For immunoblots directed to assay nuclear proteins, the nuclear extracts were obtained by using a nuclear extract kit (Active Motif). Anti-TATA-box-binding protein (Diagenode TBPCSH-100) and anti-histone H3 Ab were used for protein load control.

### Laser-Scanning Confocal Fluorescence Microscopy

Dendritic cells were seeded on poly-lysine-coated glass coverslips for 12 h and then stimulated with zymosan particles. Cells were fixed with 10% formaldehyde in PBS and stained with anti-HIF1α Ab or anti-PKM2 Ab and goat anti-rabbit IgG Ab labeled with Alexa-Fluor^®^480. The coverslips were observed by laser-scanning confocal fluorescence microscopy using a Leica TCS SP5 apparatus equipped with a white-light laser and a Leica 63PL APO NA 1.40 oil immersion objective. Image analysis and subcellular colocalization fluorograms were generated and analyzed using the Leica confocal software package LAS AF Lite and Adobe Photoshop CS5.1 software.

### Real-time RT-PCR

Total RNA was obtained by TRIzol/chloroform extraction and used for RT reactions. Cycling conditions were adapted to each set of primers. *GAPDH* was used as a housekeeping gene to assess the relative abundance of the different mRNA using the comparative cycle threshold method. The procedure was used to assay *CHOP, IL10, IL23A, IL12A, IL12B*, and *IL1B* mRNA. The sequences of the primers are shown in Table 1.

### Assay of Arachidonate Metabolites by Reversed Phase Ultraperformance Liquid Chromatography (UPLC) and Electrospray Ionization Quadrupole Time-of-Flight Mass Spectrometry (MS)

Lipids were extracted into ethanol from cell supernatants, eluted in methanol using Strata™ C-18E SPE cartridges (Phenomenex^®^), and evaporated to dryness under N_2_. The chromatographic separation was conducted in an Acquity™ UPLC System equipped with an Acquity UPLC^®^ BEH C18, 1.7 µm, 2.1 × 100 mm column (Waters). The chromatographic column was directly interfaced into the electrospray ionization source of a mass spectrometer (SYNAPT HDMS G2) from Waters. MS analysis was performed in negative ion mode using a MS^E^ method that allows simultaneous detection of analytes through a low energy function (full scan) and a high energy function (collision energy) with ion partial fragmentation, as reported (31).

### Chromatin Immunoprecipitation (ChIP) Assay

Chromatin immunoprecipitation assays were conducted with Ab against P-T71-ATF2 (Cell Signaling #9221), ATF4 (Santa Cruz sc-7583), C/EBPβ (Santa Cruz sc-150), and sXBP1 as previously reported (21). Briefly, cells were stimulated and then washed twice with PBS and fixed with 1% formaldehyde. Cross-linking was terminated by 0.125 M glycine. Crude nuclear extracts were collected by microcentrifugation and resuspended in a lysis buffer containing a high salt concentration. Chromatin sonication was carried out using a Bioruptor device from Diagenode. The chromatin solution was precleared by adding Protein A/G PLUS-Agarose for 30 min at 4°C under continuous rotation. After elimination of the beads, Ab was added for overnight incubation at 4°C, and then Protein A/G PLUS-Agarose was added and incubated for an additional period of 2 h at 4°C. Beads were harvested by centrifugation at 4,000*g* and sequentially washed with lysis buffer high salt, wash buffer, and elution buffer. Cross-links were reversed by heating at 67°C in a water bath, and the DNA bound to the beads isolated by extraction with phenol/chloroform/isoamylalcohol. Irrelevant Ab and sequences of the *IL12A* promoter were used as control of binding specificity. The sequences of the primers are shown in Table 2. Results are expressed as percentage of input.

**Table 2:** Primers used for ChIP.

*IL12A* Control FWD	5′-GCGAACATTTCGCTTTCATT-3′
*IL12A* Control REV	5′-ACTTTCCCGGGACTCTGGT-3′
*IL23A* X2-Box-C/EBP FWD	5′-CTCTAGCCACAGCAACCACA-3′
*IL23A* X2-Box-C/EBP REV	5′-GCCCGCCCTTTATACCAGCA-3′
*IL23A* X2-Box medial FWD	5′-CTTAGCTGTTTCACTCGATGTT-3′
*IL23A* X2-Box medial REV	5′-CAGGAGTTCTGGGTAGTCG-3′
*IL23A* X2-Box distal FWD	5′-TTCCATTGGTGTCCACCTTA-3′
*IL23A* X2-Box distal REV	5′-CTTTAGATTAAACATTTCCAGCA-3′
*IL23A* CHOP-C/EBP proximal FWD	5′-AGAACTCCTGGGCTTCCTAGCCAT-3′
*IL23A* CHOP-C/EBP proximal REV	5′-GGCCTCATTCTGACGTCATCCA-3′
*IL23A* CHOP-C/EBP FWD	5′-TAACGGTTTAGGCCCAGCTGAC-3′
*IL23A* CHOP-C/EBP distal REV	5′-TGTTGCGTGGCAGGAACTACA-3′
*IL23A* C/EBPβ FWD	5′-TTCCCAGTTCTCCAAGTTCC-3′
*IL23A* C/EBPβ REV	5′-TTGATTCCTACCTGATGCCC-3′
*IL23A* CRE FWD	5′-AGACCTCCATTCAGGACAAG-3′
*IL23A* CRE REV	5′-TCGAAGACGTCAGAATGAGG-3′
*IL23A* ATF2 FWD	5′-CATTGCAAACAGCTCACCAT-3′
*IL23A* ATF2 REV	5′-ATTTCCTCACTTCCTCCTGC-3′
*GFPT1* × 2-Box FWD	5′-GAGTTTCTCCCTCCCTCTC-3′
*GFPT1* × 2-Box REV	5′-GCTCCATTGAACCGCTCAC-3′

*ChIP, chromatin immunoprecipitation*.

### Statistical Analysis

Data are represented as the mean ± SEM and were analyzed with the Prism 4.0 statistical program (GraphPad Software). Comparison between experimental groups was carried out using the two-tailed Student’s *t*-test and ANOVA. Differences were considered significant for *p* < 0.05.

## Results

### Metabolic Profile of DCs

**Figure 1:**
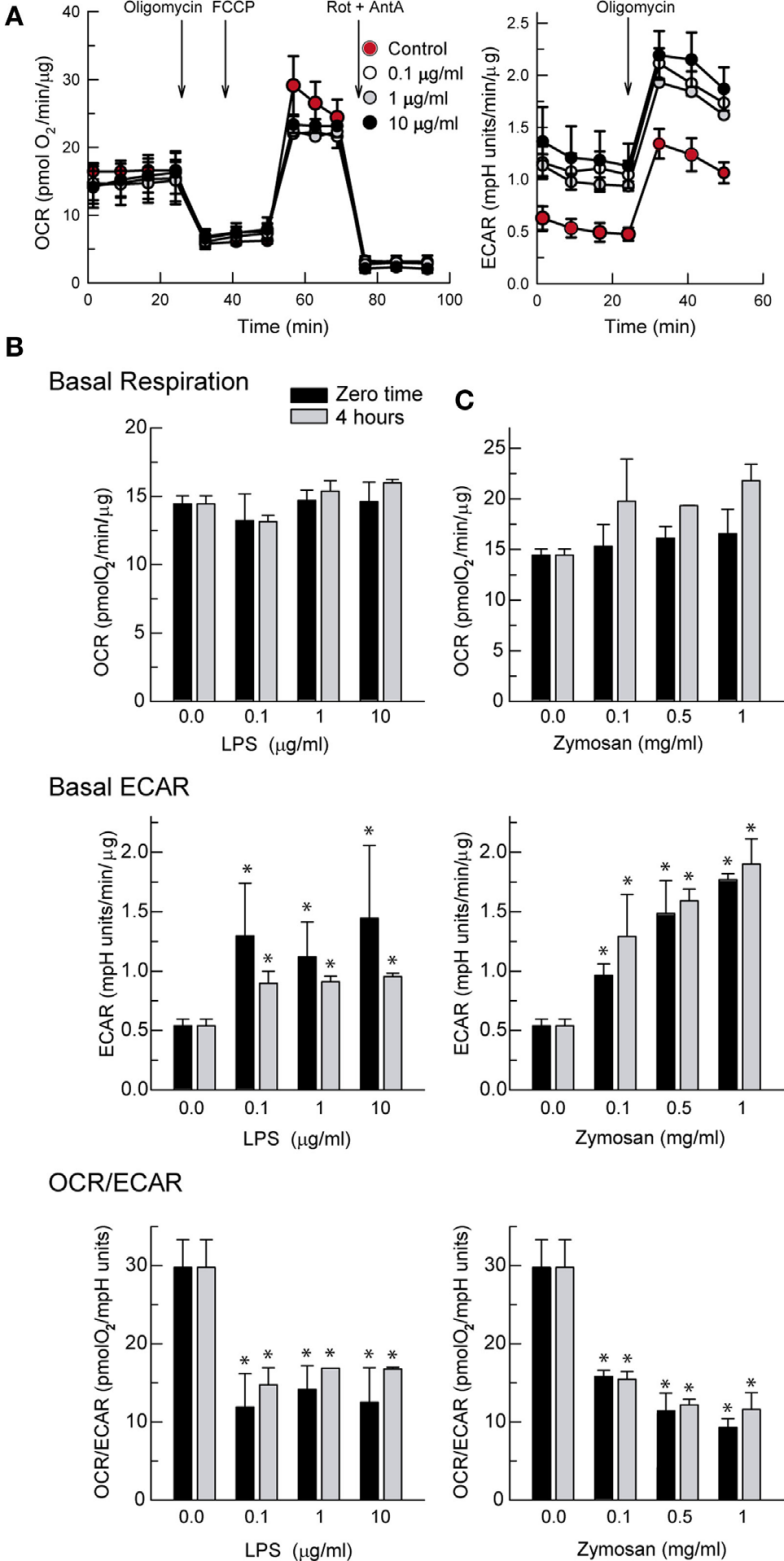
Effect of lipopolysaccharide (LPS) and zymosan on the bioenergetics of dendritic cells (DCs). **(A)** Recordings of the XF Mito Stress Test Kit protocol obtained in the presence of different concentrations of LPS. **(A–C)** DCs were stimulated for the times indicated in XF Seahorse assay medium in the presence of different concentrations of LPS and zymosan and then immediately adhered to poly-ornithine-coated plates for the assay of extracellular acidification rate (ECAR) and oxygen consumption rate (OCR). Results represent mean ± SEM of three independent experiments. *p < 0.05 as compared to DCs maintained in the absence of stimuli.

Stimulation of DCs with LPS produced a concentration-dependent increase of the ECAR that could be detected immediately after the addition of the stimuli in the XF Mito Stress Test (Figure 1A, right panel). Assessment of the OCR showed stable values in the case of LPS (Figure 1B) and a tendency to increase in the case of zymosan that did not reach statistical significance (Figure 1C). The ECAR values were somewhat higher in response to zymosan than in the case of LPS and showed a more reproducible pattern of response (Figure 1C). These results are consistent with a fast and lasting increase of glycolysis upon a wide range of PAMPs concentrations, as judged from the net decrease of the OCR/ECAR ratio (Figures 1B,C, bottom panels).

### Mechanisms Underlying Metabolic Rewiring

**Figure 2:**
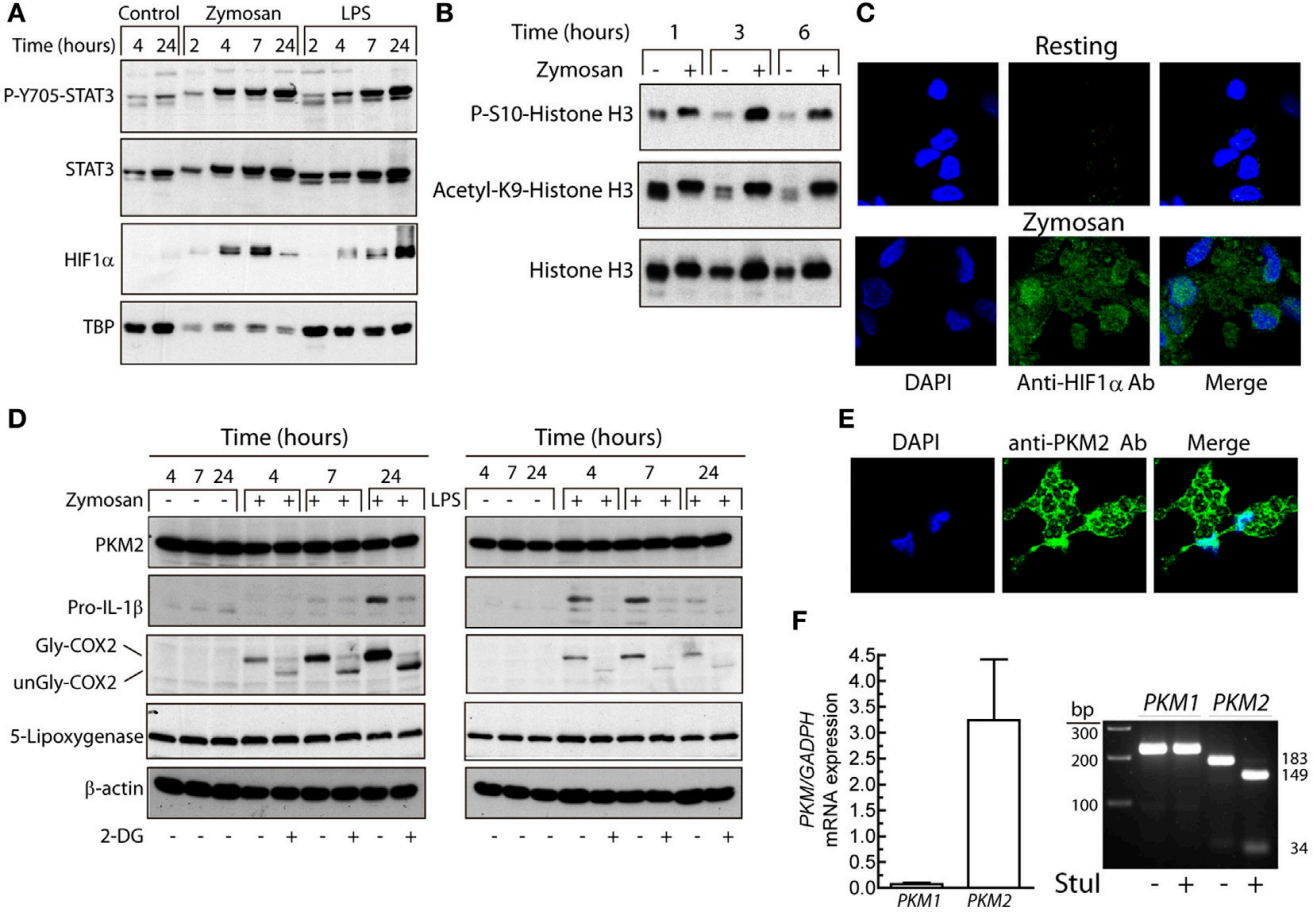
Effect of dendritic cells (DCs) stimulation on Y705-STAT3 phosphorylation and HIF1α protein expression. **(A,B)** Nuclear fractions were used for the assay of STAT3 phosphorylation, HIF1α protein, and S10-phosphorylation and K9-acetylation of histone H3. **(C)** Nuclear translocation of HIF1α assayed by laser-scanning confocal immunofluorescence microscopy in resting and zymosan-treated DCs. **(D)** The expression of pyruvate kinase M2 (PKM2), pro-IL-1β, cyclooxygenase 2 (COX-2), and 5-lipoxygenase was immunodetected in DCs stimulated with zymosan and lipopolysaccharide (LPS) for different times in DCs preincubated for 1 h in the presence or absence of 10 mM 2-deoxy-d-glucose (2-DG) prior to the addition of the pathogen-associated molecular patterns (PAMPs). **(E)** Location of PKM2 to cytoplasm and nucleus assayed by laser-scanning confocal immunofluorescence microscopy in DCs stimulated with zymosan. **(F)** Confirmation by RT-PCRs and selective digestion by *Stu*I of the *PKM2* amplicon of the predominant expression of PKM2. TBP indicates TATA-box-binding protein. Gly indicates glycosylated and unGly, unglycosylated.

One of the mechanisms promoting glycolysis in the presence of normoxia, usually termed Warburg effect or aerobic glycolysis, is the activation of HIF1 by the stabilization of its HIF1α subunit. This explains the induction of glycolytic enzyme expression, which in macrophages depends on the formation of a complex with pyruvate kinase M2 (PKM2) (32) that translocates to the nucleus and regulates the expression of proglycolytic enzymes (33). PKM2 may act as a protein kinase that phosphorylates STAT3 at Y705 (34) and histone H3 (35). This explains a positive feedback loop between HIF1 and STAT3 since Y705-STAT3 activates HIF1α transcription (36, 37). In a previous study, we observed that both zymosan and LPS are robust activators of Y705-STAT3 phosphorylation through the induction of secondary mediators (38). In keeping with that report, the assay of P-Y705-STAT3 in nuclear extracts showed a time-course compatible with its involvement in HIF1α expression (Figure 2A). As a correlate of enhanced transcriptional activity, zymosan induced global phosphorylation of S10-histone H3 and acetylation of K9-histone H3 (Figure 2B). The nuclear translocation of HIF1α was confirmed in immunofluorescence confocal microscopy studies (Figure 2C). The preferential expression of PKM2 was confirmed by Western blot (Figure 2D) and by immunofluorescence confocal microscopy, which showed the enzyme in the nuclei and in cytoplasm areas surrounding phagocytosed zymosan particles (Figure 2E), as well by analysis of its mRNA by RT-PCR. Assays with sets of primers that generate products distinguishable by the number of bp, showed a 149 and a 34 bp product in *PKM2* upon treatment with *Stu*I, whereas the *PKM1* amplicon was not digested (Figure 2F). Taken collectively, these data show the presence of P-Y705-STAT3 and HIF1α in the nuclei of zymosan- and LPS-stimulated DCs, as well as a high expression of PKM2 protein. However, the temporal pattern of HIF1α protein induction does not parallel the early changes of both OCR and ECAR, thus suggesting that other mechanisms may underlie the early rewiring of the bioenergetic profile of DCs, i.e., the association of hexokinase II with voltage-dependent anion channels in the outer mitochondrial membrane (39).

### Effect of Glucose Starvation and 2-DG on Cytokine Expression

**Figure 3:**
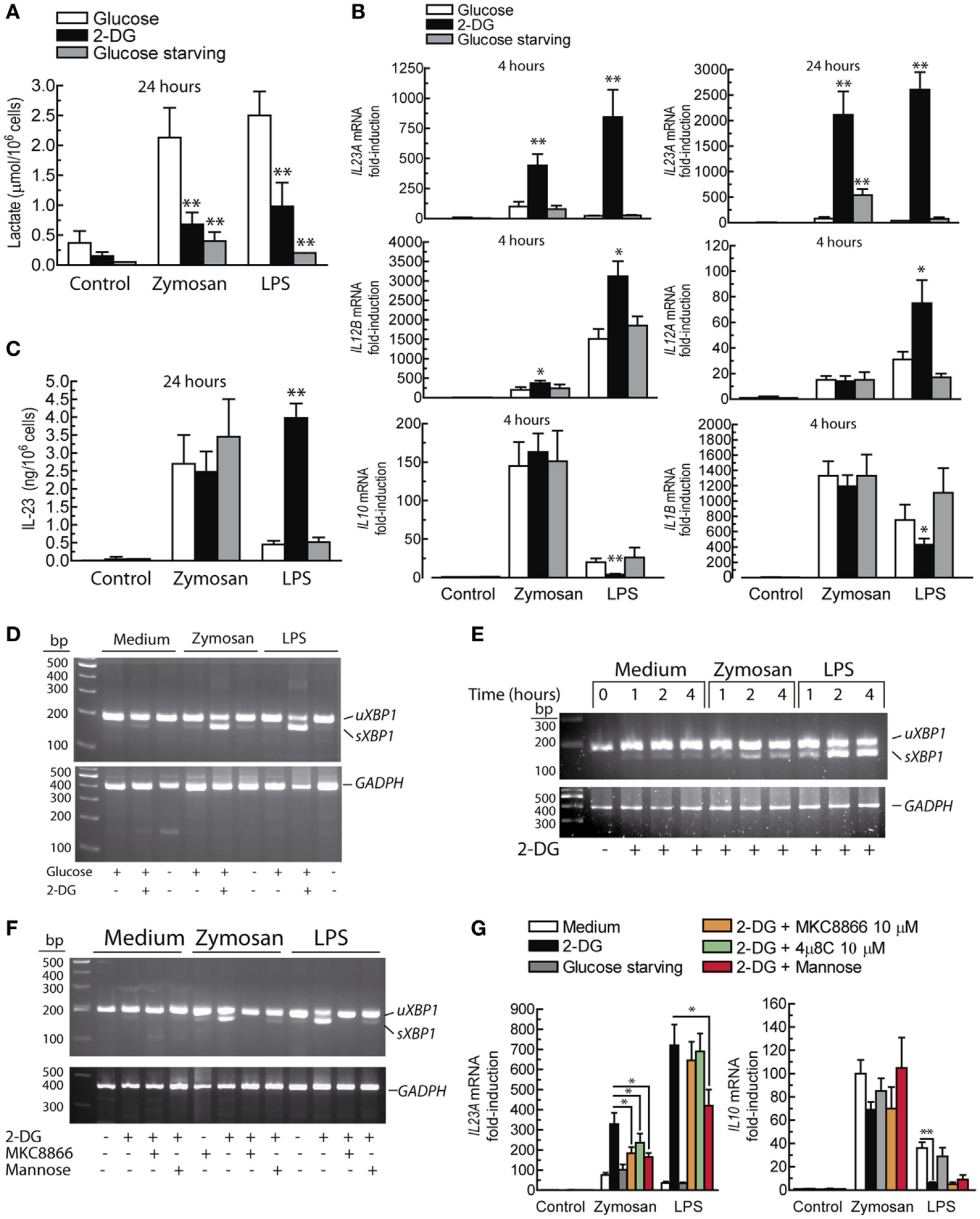
Effect of 2-deoxy-d-glucose (2-DG) on lactate production, cytokine expression, and *XBP1* splicing. **(A)** Production of lactate by dendritic cells (DCs) in the presence and absence of 11.1 mM glucose and in the presence of 10 mM 2-DG. **(B)** Effect of 2-DG or glucose starvation on the expression of the mRNA of different cytokines in DCs stimulated with 1 mg/mL zymosan and 10 µg/mL lipopolysaccharide (LPS) for the times indicated. **(C)** Effect of 2-DG on the production of IL-23 protein and on **(D)** *XBP1* mRNA splicing. **(E)** Time course of *XBP1* mRNA splicing and **(F)** effect of 1 mM mannose and 10 µM MKC8866 on the splicing of *XBP1* mRNA elicited by 2-DG. **(G)** Effect of mannose and inositol-requiring protein 1α (IRE1α) endonuclease inhibitors on the mRNA expression of *IL23A* and *IL10*. Results show mean ± SEM of 5 to 10 experiments in the mRNA assays. XBP1 mRNA splicing assays have been carried out at least twice. *sXBP1* stands for spliced X-box binding protein 1, *uXBP1* stands for unspliced *XBP1* (*p < 0.05, **p < 0.01).

**Figure 4:**
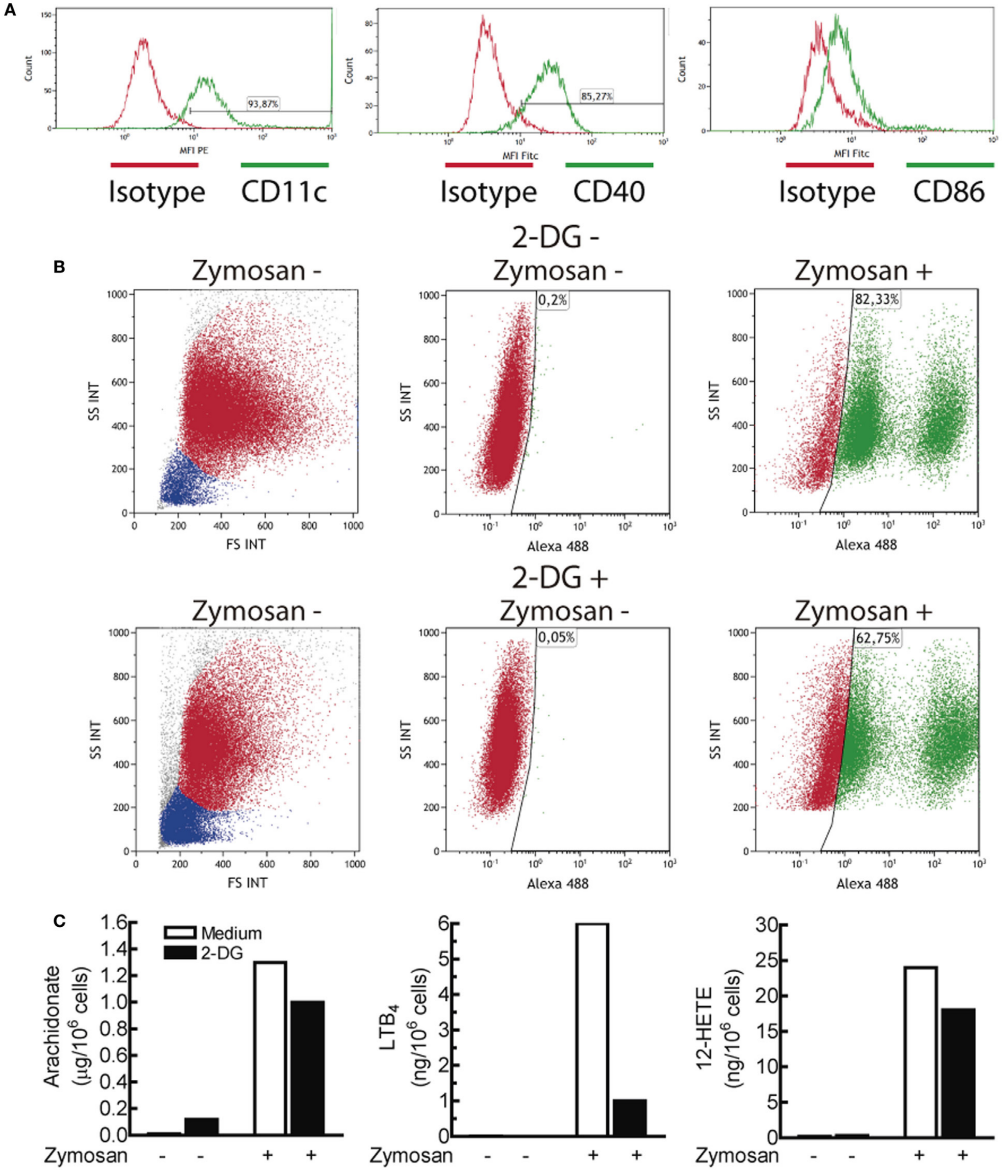
Characterization of the dendritic cell (DCs) population and effect of 2-deoxy-d-glucose (2-DG) on zymosan phagocytosis and arachidonate release. **(A)** Expression of CD11c, CD40, and CD86 in monocyte-derived DCs. **(B)** Effect of 2-DG on the phagocytosis of Alexa Fluor^®^ 488 dye-conjugated zymosan particles. DCs were incubated in the presence and absence of 10 mM 2-DG for 1 h. At the end of this period, Alexa Fluor^®^ 488-conjugated zymosan particles were added at a concentration of three particles per DC, and then the uptake of zymosan particles was assayed by flow cytometry. The dot plots represent side scatter (SS) versus forward scatter (FS) intensities in the left panels, and SS intensity versus Alexa Fluor^®^ 488 green fluorescence in the middle and right panels. **(C)** DCs were stimulated with 1 mg/mL zymosan particles for 1 h in the presence and absence of 2-DG and the supernatants collected for the assay of the most abundant eicosanoids. PGE_2_ was not detected under these conditions. LTB_4_ indicates leukotriene B_4_.

Given the increases of the ECAR induced by PAMPs, lactate levels were assayed. Lactate production was strongly blocked in glucose-starved cells and to a lesser extent in the presence of 10 mM 2-DG (Figure 3A). Consistent with seminal studies on non-opsonic phagocytosis, in which 2-DG did not reduce zymosan uptake even when ATP production was blocked (40, 41), DCs showed robust phagocytosis in the presence of 2-DG and also exhibited a robust ability to release arachidonic acid (Figure 4), thus suggesting that the effect of 2-DG cannot be explained by an effect on the earliest steps of DCs activation. Unexpectedly, the mRNA levels of various cytokines showed notable differences under glucose-limiting conditions and in the presence of 2-DG. The most striking effect was observed on the induction of *IL23A*, which increased ~ 20-fold 24 h after stimulation (Figure 3B). The assay of IL-23 protein showed a net increase in the case of LPS, but not in the case of zymosan (Figure 3C). A likely explanation is that the high production of IL-23 protein induced by zymosan under these conditions could not increase because, unlike LPS, zymosan triggers low transcription of the gene encoding the p40 chain (*IL12B*), which is a limiting step for the production of this heterodimeric cytokine. The assay of other cytokines showed prominent changes in response to LPS, including an increase of *IL12A* mRNA and a reduction of *IL10* and *IL1B* mRNA. The expression of pro-IL-1β was also blunted in the presence of 2-DG (Figure 2D). Given that these findings could not be explained by a simple blockade of glucose metabolism, alternative mechanisms were postulated, focusing on the effect of 2-DG on mannose metabolism, N-glycosylation reactions, and the possible activation of the UPR as a result of potential protein misfolding. Consistent with this hypothesis, stimulation of DCs with zymosan and LPS in the presence of 2-DG induced robust splicing of the *XBP1* mRNA (Figure 3D), whereas the sole addition of 2-DG or PAMP did not. In sharp contrast, *XBP1* splicing was not observed in DCs starved of glucose for up to 18 h and stimulated with PAMPs (not shown). Given the prominent enhancement of aerobic glycolysis induced by PAMPs and a recent report underscoring that this process reduces the supply of fructose-6-phosphate to glutamine-fructose-6-phosphate transaminase 1 (GFPT1), the enzyme that transfers the amino group of l-glutamine to yield glucosamine-6-phosphate for N-glycan biosynthesis (42), our data point out that the coincidental inhibition of mannose metabolism by 2-DG and of the hexosamine biosynthetic pathway by aerobic glycolysis show optimal conditions for N-glycosylation blockade. *XBP1* splicing was observed after 2 h of incubation (Figure 3E) and was blunted by 1 mM mannose and the inhibitor of the endonuclease activity of IRE1α MKC8866 (Figure 3F). Notably, these treatments also inhibited the effect of 2-DG on the induction of *IL23A* elicited by zymosan (Figure 3G), whereas this effect was less definite in the case of LPS. The effect of 2-DG on *IL10* mRNA induction was less sensitive to mannose and IRE1α inhibitors (Figure 3G, right panel). These data suggest that the effect of 2-DG on *IL23A* expression can be explained by interference with mannose metabolism and associated with the activation of the UPR, whereas the effect on *IL10* mRNA expression seems dependent on other mechanisms.

### Effect of Lactate

**Figure 5:**
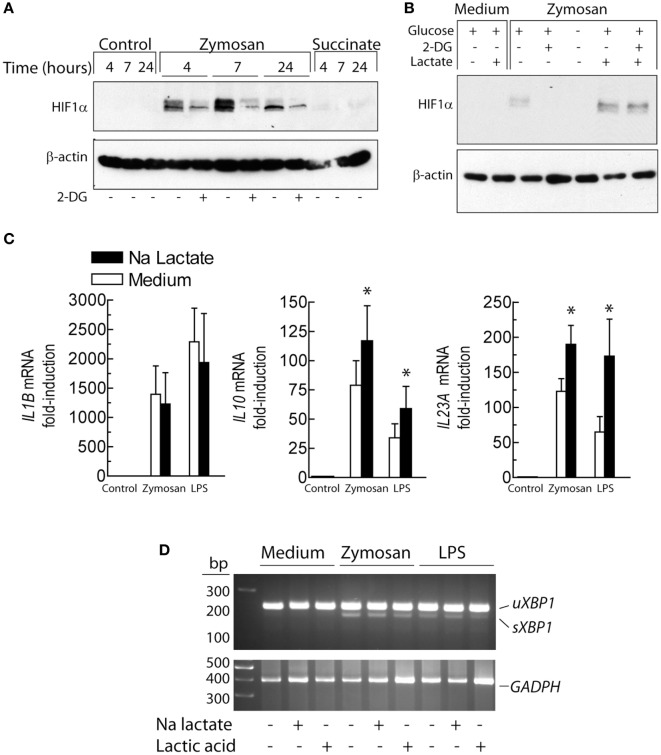
Effect of lactate and 2-deoxy-d-glucose (2-DG) on the expression of HIF1α protein and cytokines. **(A)** Effect of 2-DG on HIF1α protein expression over different times of stimulation. **(B)** Dendritic cells (DCs) were stimulated for 4 h with 1 mg/mL zymosan in the presence and absence of 2-DG and 15 mM lactate and the expression of HIF1α protein assayed. **(C)** Expression of the mRNA of several cytokines in the presence and absence of 15 mM sodium lactate. **(D)** Absence of X-box binding protein 1 (XBP1) splicing upon lactate and lactic acid treatment. Results show mean ± SEM of three experiments in **(C)** and two assays of *XBP1* mRNA splicing (*p < 0.05).

Since the distinct effects observed on *IL23A* and *IL10* mRNA expression elicited by 2-DG could not be explained by a sole mechanism and given that 2-DG reduced lactate production (Figure 3A) and blocked pro-IL-1β expression (Figure 2D), the effect of 2-DG on the lactate/HIF1 route was addressed. 2-DG inhibited the expression of HIF1α induced by zymosan (Figure 5A). An effect countered by lactate (Figure 5B). Stimulation of DCs in the presence of sodium lactate did not influence the expression of *IL1B* mRNA. In contrast, *IL10* and *IL23A* mRNA expression showed a trend to increase (Figure 5C). Treatment with LPS and zymosan in the presence of either sodium lactate or lactic acid did not induce *XBP1* splicing (Figure 5D). These results confirm the involvement of the lactate/HIF1 system in pro-IL-1β expression and show that the effect of lactate on the mRNA expression of *IL10* and *IL23A* is independent of *XBP1* splicing.

### Effect of l-Glutamine

**Figure 6:**
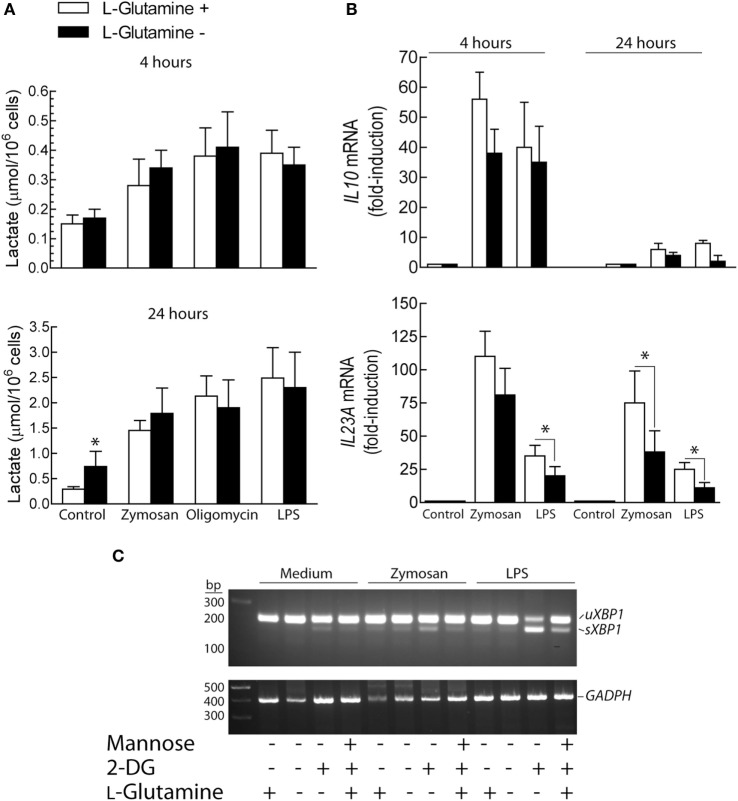
Effect of l-glutamine starvation on cytokine production. **(A–C)** Dendritic cells (DCs) were maintained for the times indicated in the presence and absence of 4 mM l-glutamine. At the end of these periods, the supernatant was collected for the assay of lactate **(A)** and the RNA extracted for the assay of the expression of cytokines **(B)** and X-box binding protein 1 (*XBP1*) splicing **(C)**. Results show mean ± SEM of three experiments (*p < 0.05).

The effect of l-glutamine starvation was addressed given its involvement in both the hexosamine biosynthesis pathway and the tricarboxylic acid cycle. In addition, glutaminolysis contributes to restore metabolic homeostasis by enhancing the XBP1- and α-ketoglutarate-dependent expression of GFPT1 (43). l-Glutamine removal induced a significant increase of lactate production in resting DCs after 24 h of incubation. In contrast, lactate levels were not significantly affected in the absence of l-glutamine after stimulation with zymosan, oligomycin or LPS (Figure 6A). *IL10* mRNA expression showed a trend to reduction in the absence of l-glutamine that reached statistical difference at 24 h (Figure 6B). Stimulation of DCs in the absence of l-glutamine did not induce *XBP1* splicing, although the effect of 2-DG was somewhat more prominent in the absence of l-glutamine (Figure 6C).

### The UPR Regulates *IL23A trans-*Activation

**Figure 7:**
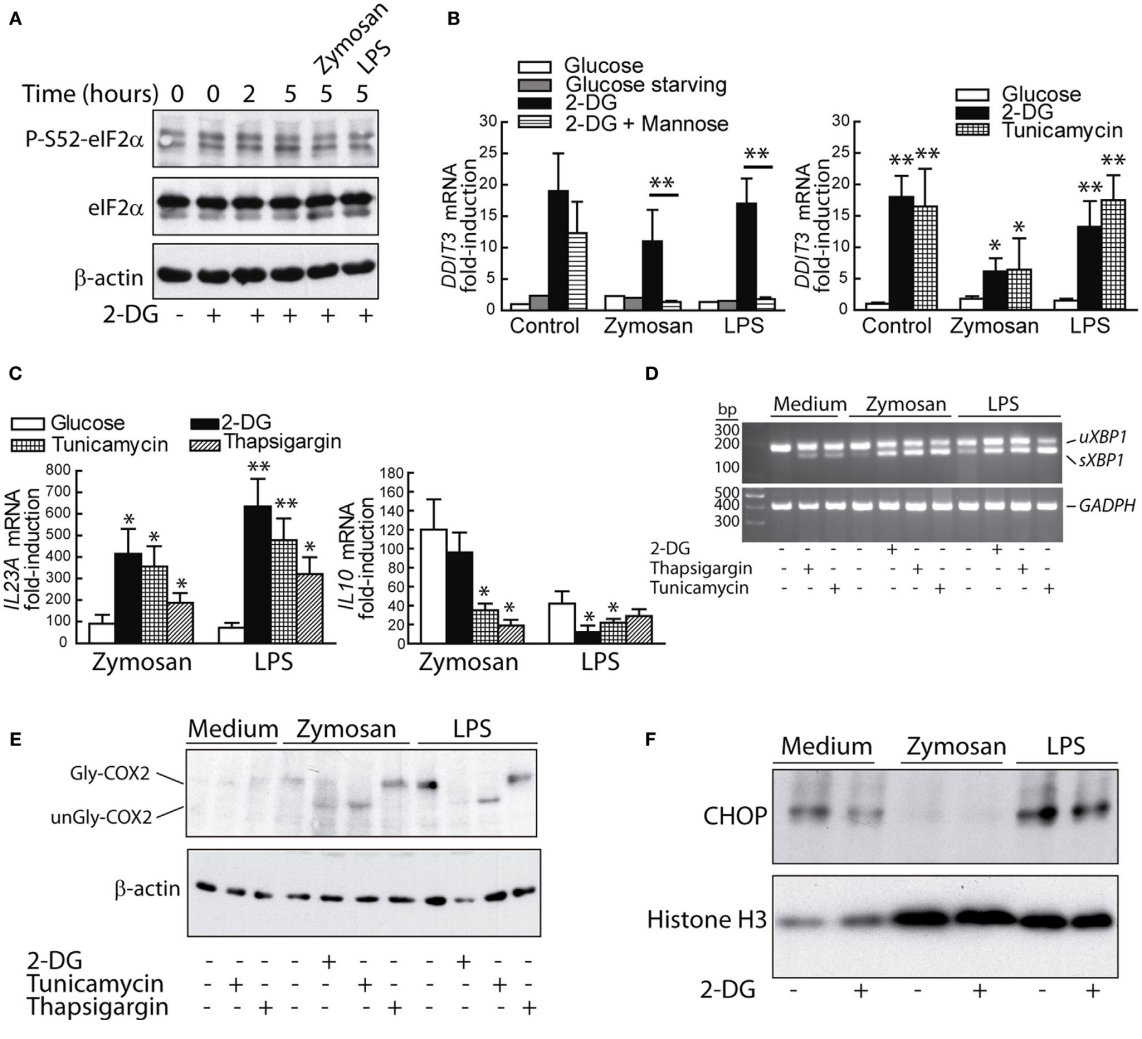

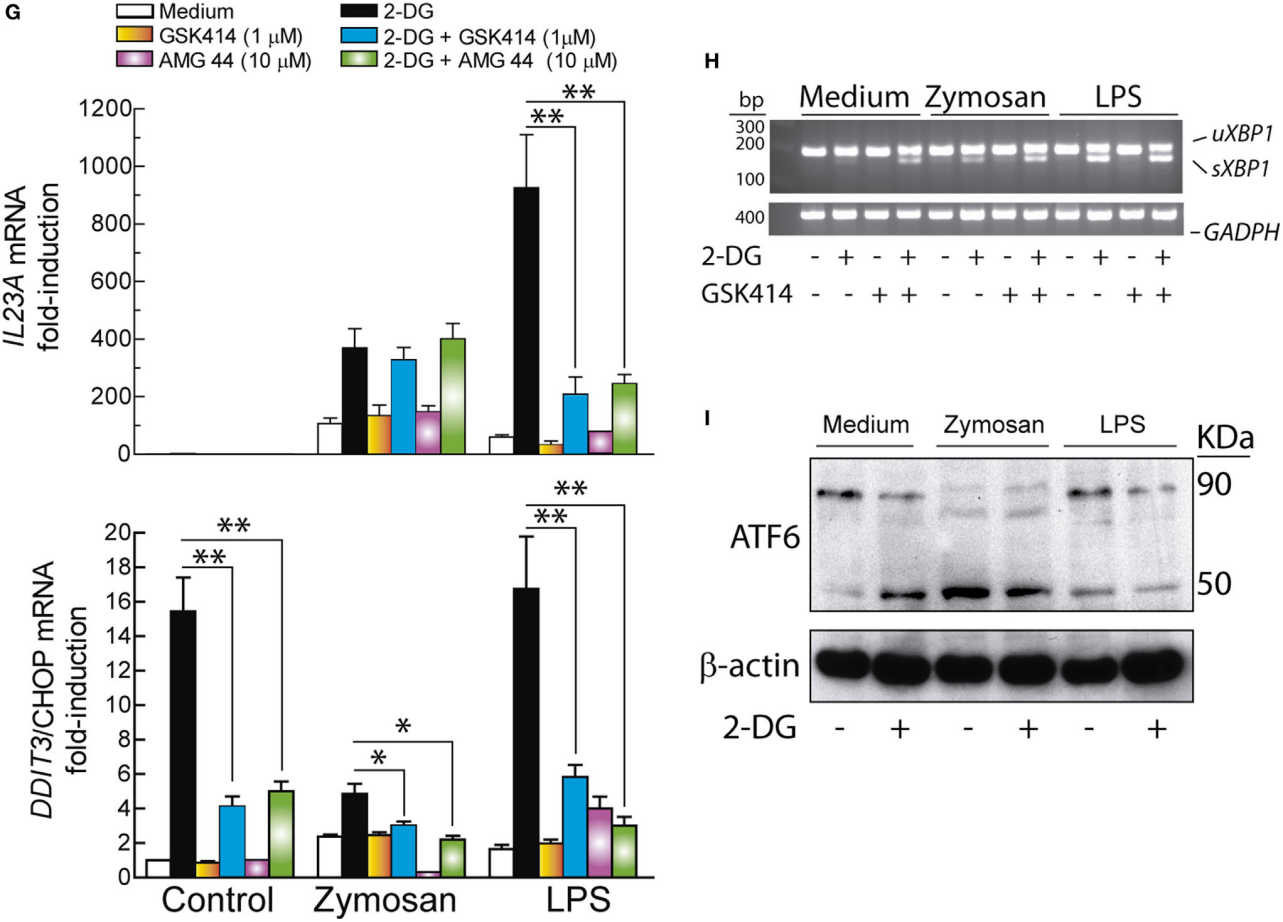
Unfolded protein response (UPR) and cytokine expression. **(A)** Time course of elongation initiation factor 2α (eIF2α) phosphorylation in dendritic cells (DCs) incubated with 2-deoxy-d-glucose (2-DG) and effect of zymosan and lipopolysaccharide (LPS) after preincubation with 2-DG for 1 h and stimulation for 4 h. **(B,C)** DCs were stimulated with LPS or zymosan for 4 h in the presence of different additions and the mRNA of *DDIT3*/C/EBP homologous protein (CHOP), *IL23A*, and *IL10* assayed. **(D)** Splicing of X-box binding protein 1 (*XBP1*) and unglycosylation of cyclooxygenase 2 (COX-2) **(E)** after 4 h of stimulation in the presence of the indicated additions. **(F)** Expression of CHOP protein in the nuclear extracts of DCs stimulated in the presence and absence of 2-DG. **(G)** Effect of 1 µM GSK2606414 and 10 µM AMG PERK 44 on *IL23A* and *DDIT3*/CHOP mRNA expression. The drugs were added 30 min prior to preincubation with 2-DG and the mRNA extracted after 4 h of incubation in the presence of the stimuli. **(H)** Lack of effect of GSK2606414 on *XBP1* splicing. **(I)** Effect of 2-DG on ATF6 cleavage in DCs maintained for 1 h in the presence or absence of 2-DG and then stimulated for 1 h with zymosan or LPS. Experiments were carried out at least in duplicate (*p < 0.05; **p < 0.01).

Given the association of *XBP1* splicing with the enhanced expression of *IL23A* mRNA, our experiments then focused on whether the UPR induced by other mechanisms showed similar results and the possible involvement of the PERK/eIF2α/ATF4/CHOP branch of the UPR. P-S52-eIF2α was detected to a low extent in resting DCs and did not increase upon 2-DG treatment and PAMPs stimulation (Figure 7A), in agreement with a recent report showing that LPS did not affect eIF2α phosphorylation (44). The mRNA expression of *DDIT3*/CHOP was enhanced by 2-DG and blunted by mannose in the presence of PAMPs (Figure 7B), thus suggesting that mannose may counter some of the mechanisms triggered by the stimuli by acting as a substrate for the formation of dolichol-linked oligosaccharide. Tunicamycin and thapsigargin induced similar effects to those elicited by 2-DG on *IL23A* and *IL10* mRNA expression (Figure 7C) as well as on *XBP1* splicing (Figure 7D). Consistent with the inhibitory activity of 2-DG on COX-2 N-glycosylation (Figure 2D), tunicamycin, a robust inhibitor of this posttranslational modification, caused the same effect. In contrast, thapsigargin, which elicits the UPR via SERCA inhibition and does not interfere with N-glycosylation reactions, failed to do so (Figure 7E). These data indicate that ER stress responses, rather than N-glycosylation inhibition alone, underpin the cytokine signature induced by ER stressors. In keeping with previous results (21), the expression of CHOP protein was detectable in the nuclear fractions of resting DCs and showed a net trend to decrease after stimulation by zymosan (Figure 7F). A recent report has disclosed that PERK inhibition decreases UPR-dependent inflammation via attenuation of eIF2α phosphorylation and reduction of the transcription of inflammatory genes as a result of translational repression (45). Consistent with this mechanism, PERK inhibition with GSK2606414 reduced the induction of *DDIT3* and *IL23A* mRNA elicited by LPS in the presence of 2-DG. A similar result was obtained with the pyrazole-based PERK inhibitor AMG PERK 44, which has been found recently to lack the RIPK1-inhibitory effect recently disclosed for GSK2606414 (30). The effect of both compounds was less evident in zymosan-treated DCs and suggests a stimulus-dependent recruitment of the different arms of the UPR (Figure 7G). Consistent with the selectivity of GSK2006414 on the PERK route, *XBP1* splicing was not inhibited (Figure 7H). Stimulation with zymosan induced ATF6 cleavage to an extent higher than that produced by the sole addition of 2-DG, as well as a noticeable change in the mobility of full-length ATF6, most likely due to the underglycosylation of the protein before cleavage by site-1 proteases (46). Combination of zymosan with 2-DG did not enhance ATF6 cleavage and the effect of LPS was similar to the one elicited by the sole addition of 2-DG (Figure 7I).

**Figure 8:**
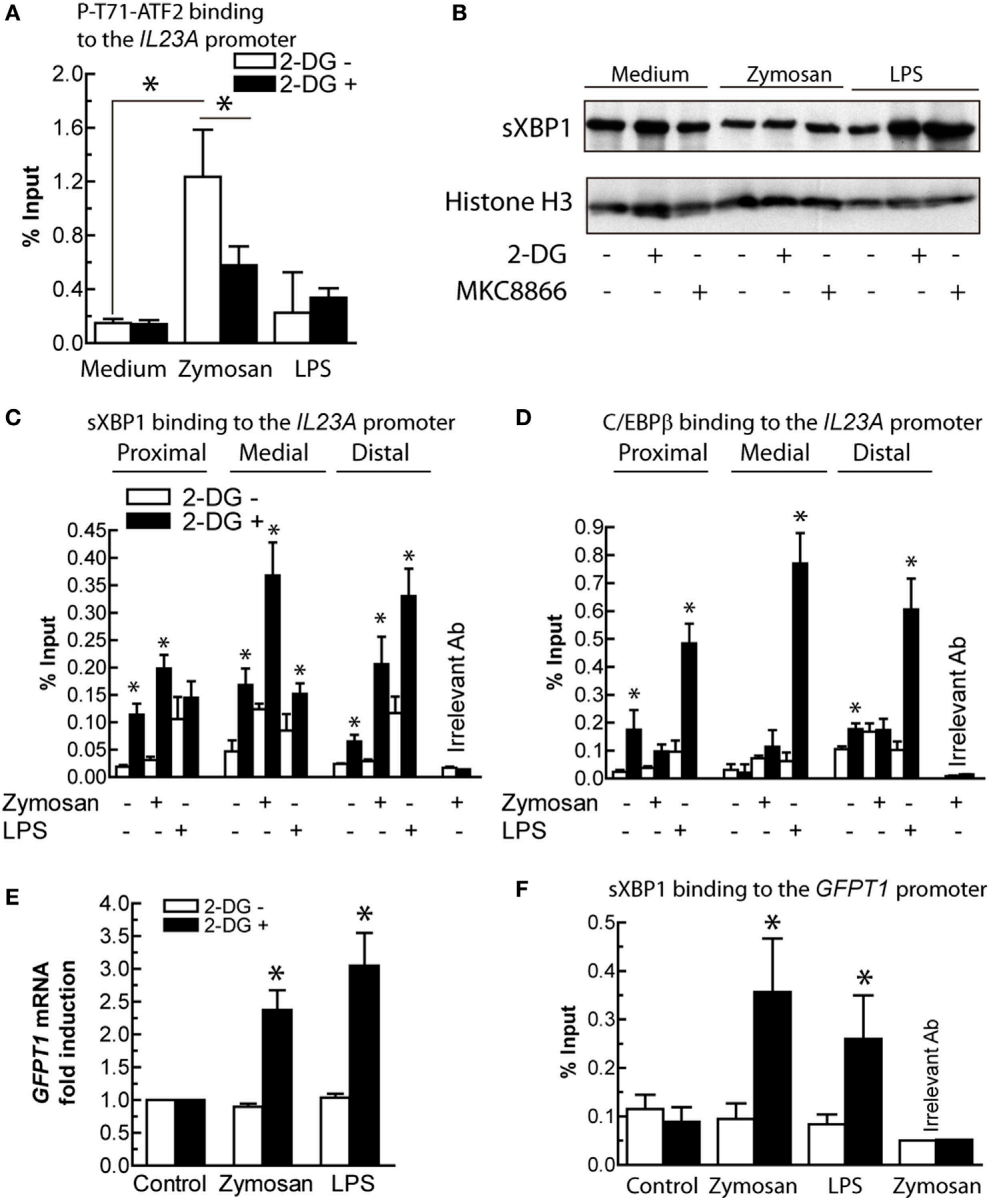
Transcription factors involved in *IL23A* and glutamine-fructose-6-phosphate transaminase 1 (*GFPT1*) mRNA expression. **(A)** Effect of 2-deoxy-d-glucose (2-DG) on the binding of P-T71-ATF2 to the ATF2 consensus binding sequence in the *IL23A* promoter. **(B)** Expression of spliced X-box binding protein 1 (sXBP1) protein in nuclear fractions of dendritic cells (DCs) stimulated with zymosan and lipopolysaccharide (LPS) in the presence and absence of 2-DG and MKC8866. **(C)** Effect of 2-DG on the binding of sXBP1 to the X-boxes of the *IL23A* promoter after 1 h of stimulation with zymosan and LPS. **(D)** Binding of CAAT/enhancer-binding protein β (C/EBPβ) to C/EBP sites in the *IL23A* promoter. **(E)** Effect of 2-DG on the mRNA expression of *GFPT1* mRNA in DCs stimulated for 4 h with zymosan and LPS. **(F)** Binding of sXBP1 to the X-box of the *GFPT1* promoter 1 h after addition of the stimuli. Results show mean ± SEM of three to five experiments (*p < 0.05).

Given that the *trans*-activation of *IL23A* induced by zymosan depends on at least NF-κB proteins and ATF2 (21), the interaction of P-T71-ATF2 with the *IL23A* promoter was assayed. The binding was partially inhibited in the presence of 2-DG, thus suggesting that other transcription factors might be engaged under these conditions (Figure 8A). The involvement of sXBP1 was then considered the leading hypothesis because of (i) the robust splicing of *XBP1* and the effect of IRE1α endonuclease inhibitors; (ii) the presence of three X-boxes in the *IL23A* promoter identified using the TRANSFAC database (21); and (iii) the role of XBP1 in the regulation of diverse targets exhibiting a wide array of sequence motifs (12, 47). sXBP1 protein was detected in the nuclear extract of DCs, but its expression did not show significant changes upon various treatments nor in the presence of MKC8866 (Figure 8B). 2-DG increased the binding of sXBP1 to the three X-boxes in the *IL23A* promoter (Figure 8C), which suggests the involvement of this transcription factor in the enhancement of *IL23A* expression and that in addition to the expression of the protein, either posttranslational modifications (48, 49) or formation of an enhanceosome with other factors may be necessary for full development of transcriptional activity (50). Since sXBP1 also plays a central role in the control of the hexosamine biosynthetic pathway by regulating the expression of GFPT1 (51), the expression of this enzyme and the binding of sXBP1 to an X-box placed 147 bp upstream of the transcription initiation nucleotide were addressed. Resting DCs showed a high expression of *GFPT1* mRNA, which was significantly enhanced when DCs were stimulated with either zymosan or LPS in the presence of 2-DG (Figure 8E). Notably, binding of sXBP1 to the X-box of *GFPT1* showed a parallel enhancement under these conditions (Figure 8F). These results confirm the presence of detectable amounts of sXBP1 protein in resting DCs in the absence of definite *XBP1* mRNA splicing and suggest that in addition to the expression of protein, posttranslational modifications or interaction with other factors may be necessary for its transcriptional activity.

### 2-DG Enhances C/EBPβ Binding Activity

*IL23A trans*-activation by LPS has been associated with a set of factors including NF-κB, cAMP response element-binding protein (CREB), and C/EBPβ. Consistent with this notion and the finding that inhibition of the IRE1α and PERK routes showed stimulus-dependent effects, the binding of C/EBPβ to three sites in the *IL23A* promoter, two of which are close to two X-boxes and to a κB-site (21), was addressed. Unlike zymosan, 2-DG increased robustly the binding of C/EBPβ to the three sites (Figure 8D), thus suggesting the association of this factor with the enhancing effect of 2-DG on the expression of *IL23A* induced by LPS.

### Effect of the Deletion of *Ern1* on *Il23a* Expression

**Figure 9:**
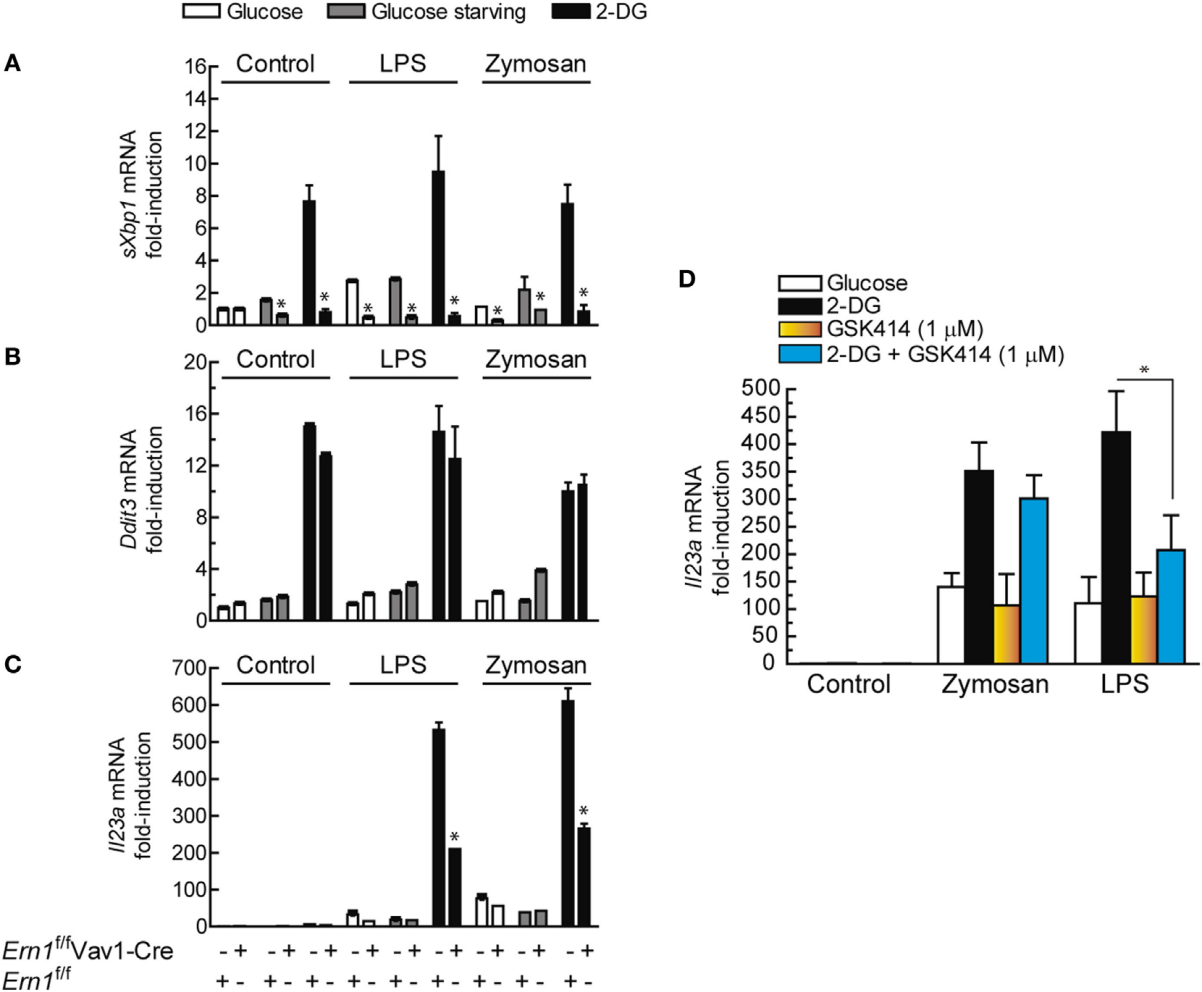
Effect of *Ern1* deletion on *Il23a* mRNA expression. **(A)** The efficiency of gene deletion in *Ern1*^f/f^Vav1-Cre was assayed by real-time RT-PCR of spliced X-box binding protein 1 (*sXBP1*) and compared with the results in *Ern1*^f/f^ mice. **(B)** Induction of *Ddit3*/Chop mRNA and **(C)** expression of *Il23a* mRNA. **(D)** C57BL/6 mice were used to address the effect of protein kinase R-like endoplasmic reticulum kinase (PERK) inhibition on *Il23a* mRNA expression. 1 µM GSK2606414 was added 30 min prior to the incubation with 2-deoxy-d-glucose (2-DG). The conditions of the experiments in bone marrow-derived dendritic cells (BMDCs) were similar to those used for dendritic cells (DCs). Results show mean ± SEM (*p < 0.05).

Since the biochemical and pharmacological experiments suggested that the effect of the UPR on *IL23A* expression could be explained by a stimulus-dependent recruitment of the IRE1α and the PERK routes, experiments were carried out in mice with hematopoietic system-specific deletion of *Ern1*, i.e., the gene encoding Ire1α (*Ern1*^f/f^Vav1-Cre mice). As shown in Figure 9A, the sole addition of 2-DG induced *Xbp1* splicing in BMDCs from *Ern1*^f/f^ mice, which is a salient difference as compared to human DCs, in which both 2-DG and PAMPs are required for *XBP1* splicing. *Ern1*^f/f^Vav1-Cre mice did not show *Xbp1* splicing, whereas *Ddit3* mRNA expression was not significantly affected (Figure 9B). Notably, a substantial reduction in *Il23a* expression was observed in the *Ern1*^f/f^Vav1-Cre mice upon treatment with both LPS and zymosan in the presence of 2-DG, as compared to the *Ern1*^f/f^ mice (Figure 9C). Consistent with the results observed in DCs, pharmacological blockade of the PERK route with GSK2606414 inhibited *Il23a* mRNA expression in WT mice stimulated with LPS (Figure 9D). These results suggest the involvement of the Ire1α route in response to both zymosan and LPS and of the PERK route in the case of LPS.

## Discussion

This study shows an increase of the extent of glycolysis along a wide array of concentrations of PAMPs and a rather stable tendency of the OCR in human DCs. The expression of HIF1α protein shows a robust increase after ~4 h, which lags behind the glycolytic changes and suggests its involvement in the maintenance of the metabolic rewiring. These results are consistent with the occurrence of an early translocation of hexokinase II to the outer mitochondrial membrane, where it takes advantage of a privileged access to mitochondrial ATP. Given the central role of hexokinase and the widespread use of its inhibitor 2-DG, we addressed the effect of glucose starvation and 2-DG on cytokine expression. Whereas glucose starvation reduced the production of lactate after DCs stimulation to levels similar to those observed in resting DCs, 2-DG produced a less intense reduction. Unexpectedly, the expression of some cytokines showed marked differences, being the effect on *IL23A* mRNA most outstanding and seemingly dependent on the IRE1α/XBP1 arm of the UPR. The different effect of 2-DG on the induction of *IL10* in response to zymosan and LPS agrees with the distinct *trans*-activating mechanisms elicited by these stimuli (38). At first glance, the effect of 2-DG could be associated with the reduction of lactate production, in keeping with the well-known function of lactate as a promoter of the differentiation of macrophages into the type M2 (3), and the observation that exogenous lactate at concentrations higher than those detected in the extracellular medium increased *IL10* mRNA. However, a correlation of lactate production with *IL10* mRNA expression could not be established since glucose deprivation was more efficient than 2-DG to reduce lactate production, while it did not affect *IL10* mRNA expression. Although an unambiguous explanation of these findings cannot be given, an effect of lactate via GPR81 is plausible, since we have observed Ca^2+^ transients by perfusing DCs with 15 mM lactate (data not shown). In addition, high concentrations of lactate have been found to induce GPR81-independent inhibition of pro-inflammatory responses and LPS-induced glycolysis in bone marrow macrophages (52). A different effect of glucose deprivation and 2-DG on cytokine production had been reported in human CD4 and CD8 T cells, although no mechanistic explanation was provided (53). A recent study has underscored the role of l-glutamine in the hexosamine biosynthesis pathway by enhancing the sXBP1- and α-ketoglutarate-dependent expression of GFPT1 via mTORC2 activation, in an attempt to restore metabolic homeostasis. However, this was only observed after very long starvation or when both l-glutamine and glucose were exhausted (43). Since the immune response entails the coordination of gene expression and protein output to avoid the risk of building-up unfolded proteins, and 2-DG has been associated with the induction of the UPR via competition with N-glycosylation reactions (24), the involvement of the transcription factors involved in the UPR was taken into account. The role of CHOP was proposed in a study carried out using a combination of LPS and tunicamycin (16), and the involvement of sXBP1 was suggested by showing that inhibition of ataxia telangiectasia mutated kinase by μM concentrations of the compound KU55933 increased the abundance of *sXBP1* mRNA and *IL23A* transcription (17). However, the experimental setting of those studies did not fit well with the actual occurrence of UPR and current views stand out the cooperation of NF-κB proteins with ATF2, CREB, and C/EBPβ as the central mechanism involved in *IL23A trans*-activation by PAMPs (20, 21, 50, 54).

**Figure 10:**
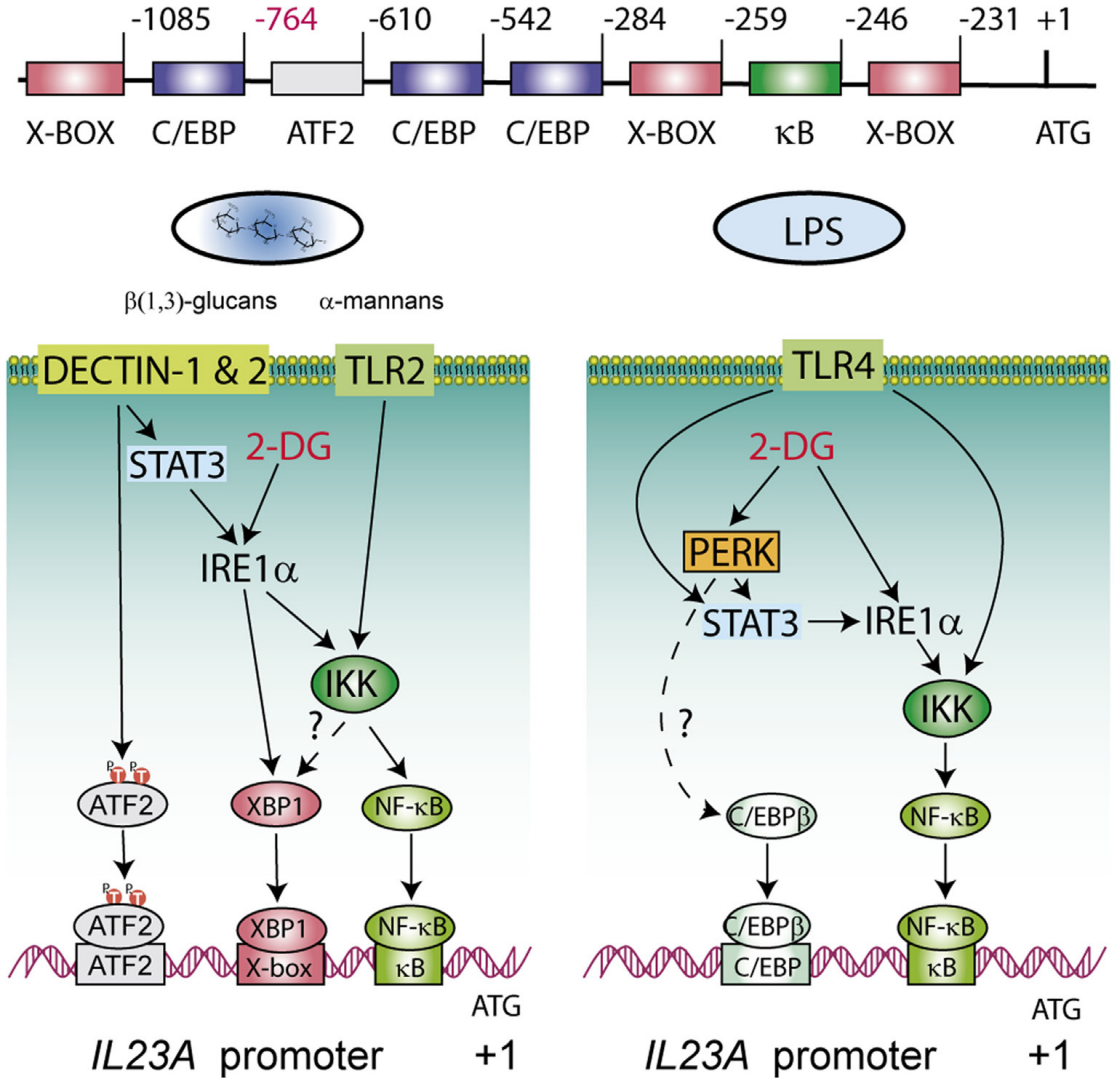
Explanatory diagram of the routes involved in the transcriptional regulation of the p19 chain of IL-23 during the unfolded protein response (UPR). The structure of the *IL23A* proximal promoter is shown on top of the diagrams underscoring the regulatory elements where activating transcription factor 2 (ATF2), X-box binding protein 1 (XBP1), NF-κB, and CAAT/enhancer-binding protein β (C/EBPβ) bind. The formation of enhanceosomes involving NF-κB, XBP1, and C/EBPβ seems most likely and the combinatory usage of elements may depend on the nature of the stimulus. The inositol-requiring protein 1α (IRE1α) route is involved in all cases and impinges at least on NF-κB activation and on XBP1 activity in the case of zymosan. STAT3 is activated by fungal patterns via secondary mediators not yet identified, whereas in the case of lipopolysaccharide (LPS) the activation depends on IFNβ and IRF3 downstream of the TRIF/TRAM adaptors of the LPS/TLR4 system. C/EBPβ and the PERK route seem more relevant in the case of LPS. Discontinuous lines indicate routes non-addressed in the current experiments but which are supported on other studies (38, 49, 59–64).

The induction of the UPR by 2-DG and tunicamycin has mechanistic commonalities since both drugs impinge on protein glycosylation. However, thapsigargin, which acts via SERCA inhibition, showed similar effects. These results indicate that the UPR rather than inhibition of N-glycosylation on its one is involved in *IL23A trans*-activation. Experiments supported the activation of XBP1 by combination of 2-DG and PAMPs and its involvement in the regulation of *IL23A* in the zymosan system. As to the PERK/eIF2α/ATF4/CHOP route, the data indicate a role of PERK in the LPS model, in agreement with the known link between translational repression and transcription of inflammatory genes (45). eIF2α phosphorylation has been associated with the enhancement of NF-κB activity via decreased levels of IκBα protein, given the short life of this protein (55). A PERK/JAK1/STAT3 route has been reported as a novel mechanism that contributes to ER stress-induced inflammation. Detection of ER stress by the ER-lumenal sensor domain dimerizes PERK and brings the interacting JAKs into proximity to allow *trans*-autophosphorylation, phosphorylation of the cytoplasmic domain of PERK at Y585 and Y619 with the ensuing activation of its kinase activity, and S52-eIF2α and Y705-STAT3 phosphorylation (56). Notably, P-Y705-STAT3 can be detected in the DCs system at early times as a result of the residual effect of the cytokines GM-CSF and IL-4 used to induce DCs differentiation (57), and later on as a result of the TRIF/TRAM branch of the LPS/TLR4 system or through the induction of secondary mediators (38). The interplay between the PERK and IRE1α routes through STAT3 (Figure 10) is supported by recent data showing that STAT3 plays a critical role in the activation of a non-canonical UPR cascade involving Ire1α and Xbp1 under combined cytokine stimulation (58). Our finding of a stable phosphorylation of eIF2α (Figure 7A) agrees with a recent report showing similar results in M2-polarized macrophages stimulated with LPS, thus suggesting that some stimuli activate the UPR in the absence of a stimulatory effect on eIF2α phosphorylation (44).

Further mechanistic insight was provided by experiments in the Ire1α-deficient mice, by showing that a significant part of the effect could be associated unambiguously with the IRE1α route. In the case of zymosan it also involved sXBP1, although other mechanisms cannot be ruled out, e.g., the formation of a complex of IκB kinase (IKK) with IRE1α through the adaptor protein TRAF2, given that NF-κB activation is impaired under ER stress conditions in IRE1α knockdown cells and *Ern1*^−/−^ mouse embryonic fibroblasts (59). A recent study has enlarged the reciprocity of connections between IKK and the IRE1α route by showing that IKKβ directly phosphorylates sXBP1 at T48 and S148 residues (49). Surprisingly, another study has unveiled a role of XBP1 as a moonlighting protein, since its antilipogenic activity is mediated through a protein-protein interaction that ultimately reduces cytoplasm-to-membrane translocation of PKCε (60).

Our suggestion that the PERK branch is involved in response to LPS mainly stems on pharmacological studies. Given that GSK2606414 has been recently reported to be an active inhibitor of RIPK1 in TNFα-RIPK1 kinase-dependent models, we extended the experiments using AMP PERK 44 (30), which does not exhibit that effect. Results are consistent with PERK activity inhibition and do not suggest RIPK1 involvement in *IL23A* induction. An open question is the association of C/EBPβ with the UPR. C/EBPβ has been found to induce the transcriptional activation of *XBP1* (61, 62) and, conversely, C/EBPβ is induced by XBP1 via an X-box present in human cells that is absent in rats and mice (63), as well as through the PERK route (64). In addition, C/EBPβ is fully active in unstimulated macrophages and poised to be recruited into enhanceosomes to team up with other factors to stimulate transcription, given its well-known function as a pioneer factor that promotes the opening of silent chromatin (49, 65, 66). The present results are of clinical relevance because DCs in tumor milieus show an activation of XBP1 that blunts the immune response and favors tumor cell progression (13). This agrees with a previous report where the poor prognosis of triple-negative breast cancers correlated with the assembly of a transcriptional complex of XBP1 and HIF1α (67). The present study extends the clinical relevance of those reports by showing that the stimulation by PAMPs under conditions of ER stress imprints the cytokine response into a pattern favorable to tumor progression. In fact, the production of IL-23 by tumor-associated myeloid cells in tissues accessible to microbial products is a major driver of tumor growth, for instance in colon cancer (15). Noteworthy, GM-CSF exerts a robust effect on atherogenesis via the production of IL-23 (68) and a recent report has shown that inhibition of the ribonuclease activity of Ire1α by 4μ8C counteracts atherosclerosis progression (69). Therefore, a major contribution of Ire1α to atherogenesis may rely on its ability to enhance IL-23 production. Most recent reports have confirmed in clinical studies the beneficial effect of blocking IL-23 activity in psoriasis (70) and Crohn’s disease (71). Most remarkable, increasing target selectivity with risankizumab, an antibody reactive to the p19 chain, induced clinical responses superior to those associated with ustekinumab, an antibody reactive to the p40 chain, which is shared by both IL-12 p70 and IL-23 (70). In summary, these data show a net enhancement of glycolysis in DCs stimulated with PAMPs. The use of the hexokinase inhibitor 2-DG disclosed robust changes of *IL23A* transcription that were not reproduced by glucose starvation and pointed to the activation of the UPR via interference with mannose metabolism and N-glycosylation reactions. These data indicate that the current mechanism of transcriptional regulation of *IL23A* induced by PAMPs via the recruitment of NF-κB, ATF2, and C/EBPβ can be reinforced by the recruitment of the IRE1α and PERK arms of the UPR. An explanatory diagram of the routes involved is shown in Figure 10.

## Ethics Statement

The study was approved by the Bioethical Committee of the Spanish Council of Research (CSIC) and the written informed consent of all healthy donors was obtained at Centro de Hemoterapia y Hemodonación de Castilla y León Biobank. The participants received written consent according to the regulations of the Biobank and the researchers received the samples in an anonymous way. The process is documented by the Biobank authority according to the specific Spanish regulations. The animal experiments were carried out with permission of the local authority and conform to institutional standards. The ethics committee approved this procedure before starting the study.

## Author Contributions

SM, JF, SA, ET-C, CH, OM, and AA conducted experiments and drafted the work. TI, JC-R, NF, and MC designed the experiments and interpreted the data. All authors reviewed and approved the manuscript. JC-R and MC wrote the article.

## Conflict of Interest Statement

JC-R is a co-founder and scientific advisor for Quentis Therapeucis Inc. The role of this author was to design experiments, analyze data, provide reagents and materials, and write the manuscript. All other authors declare no competing interests.

## Abbreviations

ATF, activating transcription factor; BMDCs, bone marrow-derived dendritic cells; C/EBPβ, CAAT/enhancer-binding protein β; ChIP, chromatin immunoprecipitation; CHOP, C/EBP homologous protein; COX-2, cyclooxygenase 2; CREB, cAMP response element-binding protein; DCs, dendritic cells; 2-DG, 2-deoxy-d-glucose; ECAR, extracellular acidification rate; eIF2α, elongation initiation factor 2α; ER, endoplasmic reticulum; GFPT1, glutamine-fructose-6-phosphate transaminase 1; HIF, hypoxia-inducible factor; IRE1α, inositol-requiring protein 1α; IKK, IκB kinase; MS, mass spectrometry; OCR, oxygen consumption rate; PAMPs, pathogen-associated molecular patterns; PERK, protein kinase R-like endoplasmic reticulum kinase; PKM, pyruvate kinase M; UPLC, ultraperformance liquid chromatography; UPR, unfolded protein response; WT, wild type; XBP1, X-box binding protein 1.
